# Composite dietary antioxidant index and the risk of heart failure: A cross‐sectional study from NHANES

**DOI:** 10.1002/clc.24144

**Published:** 2023-09-08

**Authors:** Yazhe Ma, Jiangwen Liu, Jianying Sun, Yanju Cui, Peng Wu, Feiyu Wei, Xiaolong Gao, Tao Ma, Xi Zhang, Xiaohui Kuang, Jie Fan

**Affiliations:** ^1^ Division of Cardiology, Yunnan Arrhythmia Research Center, The First People's Hospital of Yunnan Province The Affiliated Hospital of Kunming University of Science and Technology Kunming People's Republic of China; ^2^ Department of Cardiology Renmin Hospital of Wuhan University Wuhan City People's Republic of China; ^3^ Cardiovascular Research Institute of Wuhan University Wuhan City People's Republic of China; ^4^ Hubei Key Laboratory of Cardiology Wuhan City People's Republic of China

**Keywords:** composite dietary antioxidant index, heart failure, healthy diet, oxidative stress

## Abstract

**Background:**

Previous studies show that oxidative stress is important in heart failure (HF) pathogenesis. The composite dietary antioxidant index (CDAI), which reflects the antioxidant profile of nutrient supplements, is associated with cardiovascular mortality risk. However, the association between CDAI and the risk of HF remains unknown.

**Hypothesis:**

In this study, we investigated the relationship between CDAI and HF risk using National Health and Nutritional Examination Survey (NHANES) data.

**Methods:**

The data of participants aged >40 years old from the NHNANES between 2001 and 2018 were obtained and used to assess the relationship between CDAI and the risk of HF. Logistic regression was used to calculate the odds ratio (OR) of CDAI for the risk of HF.

**Results:**

A total of 29 101 participants were divided into the HF (*n* = 1419; 4.88%) and non‐HF groups (*n* = 27 682; 95.12%), HF group participants had lower CDAI than the non‐HF group (−0.32 ± 0.14 vs. 0.67 ± 0.05, *p* < .0001). Compared with the lowest CDAI quartile (Q1), the OR for HF risk was 0.88 (0.68−1.13) for Q2 (*p* = .30), 0.77 (0.61−0.99) for Q3 (*p* = .04), and 0.68 (0.52−0.89) for Q4 (*p* = .01).

**Conclusions:**

CDAI was negatively associated with the risk of HF. Our findings show that the intake of an antioxidant‐rich dietary is a potential method to reduce the risk of HF.

## Graphical abstract

This study assessed the association between composite dietary antioxidant index (CDAI) and the prevalence of heart failure (HF) in adults aged more than 40 years old in the United States, using data from National Health and Nutritional Examination Survey 2009−2018. To conclude that CDAI was negatively associated with HF risk in the population (odds ratio: 0.96, *p* = .02).

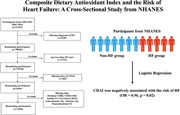

## 1 INTRODUCTION

Heart failure (HF) affects nearly 6.5 million adults in the United States, and the number of HF cases keeps increasing because of the growing and aging population.^1^, ^2^ Increasing evidence has revealed that dietary supplementation patterns are associated with HF progression. Dietary supplementation patterns, such as sodium and fluid restriction, low dietary inflammatory patterns, and Mediterranean dietary patterns can reduce the risk of HF.^3^, ^4^ It is well recognized that oxidative stress is involved in the progression of HF and that the diet‐related oxidative stress should not be neglected in cardiovascular diseases.^5^, ^6^

The composite dietary antioxidant index (CDAI) is a score reflecting the antioxidant profile of nutrient supplements. It correlates with inflammation biomarkers such as tumor necrosis factor‐α and interleukin‐1.^7^, ^8^, ^9^ A previous study has shown that a high CDAI is linked to a low cardiovascular mortality risk.^10^ However, the effects of combined dietary antioxidants on the risk of HF remains unknown. Therefore, this study aimed to investigate the relationship between CDAI and HF risk using National Health and Nutritional Examination Survey (NHANES) data.

## 2 METHODS

### 2.1 Study population

We obtained data from the NHANES conducted between 2001 and 2018. NHANES was a cross‐sectional survey to evaluate the health and nutritional status of the noninstitutionalized US population. This survey collected information on the nutritional and health statuses of American families through questionnaires survey and laboratory examinations. Details are available at https://www.cdc.gov/nchs/nhanes/about_nhanes.htm. This cross‐sectional study collected the data of patients aged >40 years old from the NHANES.

We used the “MCQ160B” variable in the questionnaire to diagnose HF, which asked if a doctor had ever diagnosed the patient with congestive HF. We selected 29 107 of the 50 042 participants aged over 40 years old after excluding those with missing CDAI data. The survey was approved by the National Center for Health Statistics Ethical Review Board, and all participants provided informed consent. Researchers can access the database without applying.^11^

### 2.2 Calculation of CDAI

Each participants' food and nutrient intake in the NHANES was collected from the dietary interview component, which called “what we eat in America.” The data analysis was conducted by the Food Surveys Research Group of the US Department of Agriculture. The participants responded to two recall surveys, the first time was in the Mobile Examination Center, while the second was on the telephone. we used the first recall data to avoid memory drift caused by too long a time. Based on the interview, we determined the intake of dietary supplements, including dosage, frequency, and duration of consumption. CDAI is based on the dietary intake of six antioxidants: zinc, selenium, carotenoids, vitamins A, C, and E.^12^

The calculation of CDAI was based on the concentration of antioxidant components in foods and the antioxidant capacity weight of each component. The total score for each food was the weighted average of all the antioxidant component scores in that food. Then, the CDAI for each participant was the weighted average of the total antioxidant capacity (TAC) scores for all food consumptions.

### 2.3 Study variables

We included specific demographics, physical examinations, and questionnaire data as covariates. Demographic variables included age, gender (male, female), education level (<high school, high school, >high school),^11^ and poverty degree (<1.2, >1.2).^13^ Questionnaire data included smoking status (never, former, now)^11^ and drinking history (never, former, now).

Diagnosing of the following covariables was based on a questionnaire or laboratory examination data. Hypertension was diagnosed when a participant was told by a doctor or when they had a blood pressure >140/90 mmHg, or a history of taking antihypertensive drugs. Metabolic syndrome (MetS) was diagnosed based on clinical judgment and required at least three of the following criteria to be met: waist circumference, blood pressure, blood glucose, triglycerides (TG), and HDL cholesterol levels.^14^ The specific criteria for each of these factors may vary according to sex and ethnicity, and physicians should consult the most recent guidelines and studies when making a diagnosis.

Hyperlipidemia^15^ was diagnosed using two methods: first, a history of lipid‐lowering drug use; and second and detecting elevated levels of serum total cholesterol (TC), low‐density lipoprotein cholesterol (LDL‐C), or TG in untreated patients. Specifically, hyperlipidemia was diagnosed when the serum TC exceeded 5.2 mmol/L, LDL‐C exceeded 3.4 mmol/L, or TG exceeded 1.7 mmol/L. Chronic kidney disease (CKD) was identified by either an estimated glomerular filtration rate <60 mL/min/1.73 m^2^ or a urine albumin−creatinine ratio >30.^16^

### 2.4 Statistical analysis

Continuous variables are presented as means ± standard error, while categorical variables are presented as quantities (percentages). An unpaired *t*‐test was used to analyze continuous variables and outcomes, and the *χ* ^2^ test was used to classify variables. Univariate logistic regression was used to analyze the association between CDAI and covariates of CDAI and covariates of HF. We used three weighted logistic regression models to evaluate the relationship between the CDAI and HF. Model 1 did not adjust for covariates, whereas Model 2 adjusted for age, gender, education, and poverty. Model 3 was adjusted all covariates. All the analyses were performed using the R software. Statistical significance was defined as a two‐tailed *p*‐value of <.05.

## 3 RESULTS

### 3.1 Participants characteristics

**Figure 1:**
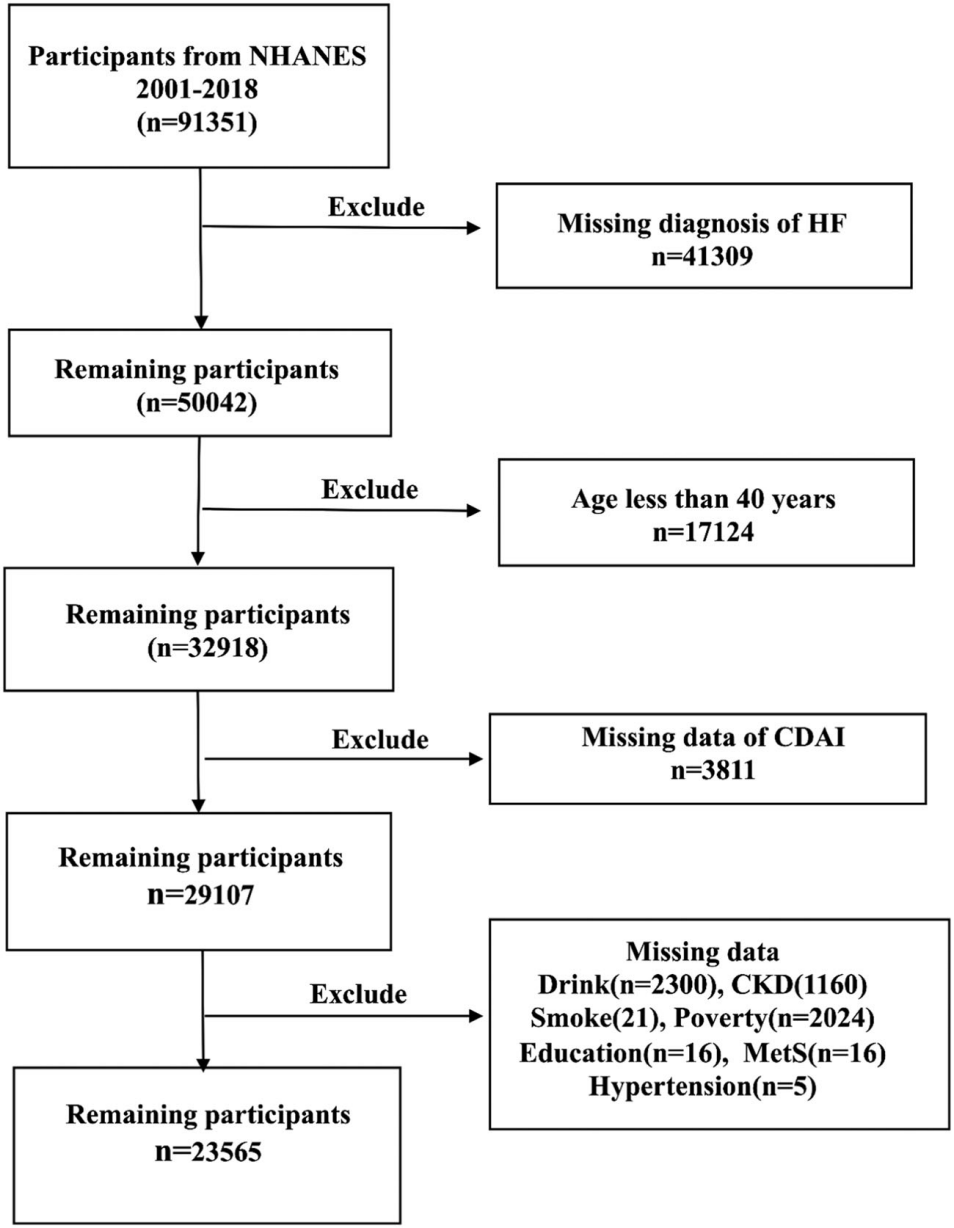
Flow chart of study participants. Sample selection and exclusion criteria for the comparison of HF and non‐HF participants. CDAI, composite dietary antioxidants index; CKD, chronic kidney disease; HF, heart failure; NHANES, national health and nutritional examination survey.

After excluding participants without an HF diagnosis, and those with missing dietary information for CDAI calculation, 40 380 participants were included from NHANES during 1999−2018. Then, they were then divided into two groups: the HF group (*n* = 1419) and the non‐HF group (*n* = 27682) (Figure 1). CDAI scores ranged from −7.352 to −2.29, −2.29 to −0.331, −0.331 to 2.132, and 2.132 to 78.016 were in quartiles1 (Q1), 2 (Q2), 3 (Q3), and 4 (Q4), respectively. The participants characteristics are listed in Table 1.

**Table 1:** Characteristics of participants from HF and non‐HF group.

Variables	Total	HF		*p* Value
		No	Yes	
Age	57.52 ± 0.15	57.14 ± 0.15	67.43 ± 0.47	<.0001
Gender				<.001
Female	11 823 (50.17)	11 347 (95.97)	476 (4.03)	
Male	11 742 (49.83)	11 085 (94.40)	657 (5.60)	
Education				<.0001
<High school	6277 (26.64)	5861 (93.37)	416 (6.63)	
High school	5487 (23.28)	5200 (94.77)	287 (5.23)	
>High school	11 801 (50.08)	11 371 (96.36)	430 (3.64)	
Smoke				<.0001
Never	11 744 (49.84)	11 317 (96.36)	427 (3.64)	
Former	7433 (31.54)	6956 (93.58)	477 (6.42)	
Now	4388 (18.62)	4159 (94.78)	229 (5.22)	
Drink				<.0001
Never	3408 (14.46)	3227 (94.69)	181 (5.31)	
Former	5183 (21.99)	4751 (91.67)	432 (8.33)	
Now	14 974 (63.54)	14 454 (96.53)	520 (3.47)	
Hypertension				<.0001
No	10 329 (43.83)	10 153 (98.30)	176 (1.70)	
Yes	13 236 (56.17)	12 279 (92.77)	957 (7.23)	
MetS				<.0001
No	13 830 (58.69)	13 421 (97.04)	409 (2.96)	
Yes	9735 (41.31)	9011 (92.56)	724 (7.44)	
Hyperlipidemia				<.0001
No	4807 (20.40)	4661 (96.96)	146 (3.04)	
Yes	18 758 (79.60)	17 771 (94.74)	987 (5.26)	
Poverty				<.0001
<1.2	5551 (23.56)	5177 (93.26)	374 (6.74)	
≥1.2	18 014 (76.44)	17 255 (95.79)	759 (4.21)	
CKD				<.0001
No	17 687 (75.06)	17 246 (97.51)	441 (2.49)	
Yes	5878 (24.94)	5186 (88.23)	692 (11.77)	
CDAI	0.69 ± 0.05	0.73 ± 0.05	−0.38 ± 0.16	<.0001
CDAIQ				<.0001
Q1 (−7.352 to −2.29)	5893 (25.01)	5503 (93.38)	390 (6.62)	
Q2 (−2.29 to −0.331)	5890 (24.99)	5574 (94.63)	316 (5.37)	
Q3 (−0.331 to 2.132)	5891 (25.00)	5660 (96.08)	231 (3.92)	
Q4 (2.132 to 78.016)	5891 (25.00)	5695 (96.67)	196 (3.33)	

Abbreviations: CDAI, composite dietary antioxidants index; CKD, chronic kidney disease; HF, heart failure; MetS, metabolic syndrome.

There were 799 (53.48%) and 14 269 (49.02%) male participants with mean ages of 67.84 ± 0.41 and 57.38 ± 0.14 years old in the HF and non‐HF groups, respectively. The HF group participants had a lower income and education than the non‐HF group (*p* < .0001). The prevalence of hypertension, MetS, hyperlipidemia, and CKD was higher in the HF group than in the non‐HF group (all *p* < .0001). More participants in the HF group were smokers and drinkers than those in the non‐HF group (*p* < .0001).

### 3.2 Association between HF and CDAI

The unadjusted and adjusted models are presented in Tables 2 and 3, respectively. Model 1 was the crude model, which showed that the odds ratio (OR) of HF were decreased with higher CDAI scores (OR [95% confidence interval [CI]] = 0.93 [0.90−0.95], *p* < .0001). After adjusting for sex, age, education, and poverty, the OR in Model 2 was 0.95. Model 3 was further adjusted for smoking, drinking, hypertension, MetS, hyperlipidemia, and CKD comparied to Model 2. There was an association between higher CDAI scores and the decreased of HF incidence (OR [95% CI] = 0.96 [0.94−0.99], *p* = .02). Compared to the lowest CDAI quartile (Q1), the OR for HF risk was 0.88 (0.68−1.13) for Q2 (*p* = .30), 0.77 (0.61−0.99) for Q3 (*p* = .04), and 0.68 (0.52−0.89) for Q4 (*p* = .01).

**Table 2:** Weighted associations between the CDAI and the risk of HF in the multivariable.

Variables	OR (95% CI)	*p* Value
Age	1.05 (1.04−1.06)	<.0001
Gender		
Female	1.00 (ref)	
Male	1.56 (1.31−1.85)	<.0001
Education		
<High school	1.00 (ref)	
High school	0.94 (0.74−1.19)	.62
>High school	0.85 (0.68−1.06)	.14
Smoke		
Never	1.00 (ref)	
Former	1.48 (1.21−1.80)	<.001
Now	2.12 (1.65−2.72)	<.0001
Drink		
Never	1.00 (ref)	
Former	1.44 (1.12−1.85)	.005
Now	0.73 (0.55−0.96)	.03
Hypertension		
No	1.00 (ref)	
Yes	2.11 (1.65−2.70)	<.0001
MetS		
No	1.00 (ref)	
Yes	1.85 (1.53−2.24)	<.0001
Hyperlipidemia		
No	1.00 (ref)	
Yes	1.30 (1.05−1.61)	.02
Poverty		
<1.2	1.00 (ref)	
≥1.2	0.66 (0.55−0.79)	<.0001
CKD		
No	1.00 (ref)	
Yes	2.53 (2.14−2.99)	<.0001
CDAI		
Q1 (−7.352 to −2.29)	1.00 (ref)	
Q2 (−2.29 to −0.331)	0.90 (0.70−1.16)	.42
Q3 (−0.331 to 2.132)	0.77 (0.60−0.99)	.04
Q4 (2.132 to 78.016)	0.68 (0.52−0.90)	.01

Abbreviations: CDAI, composite dietary antioxidants index; CI, confidence interval; CKD, chronic kidney disease; MetS, metabolic syndrome; OR, odds ratio.

**Table 3:** Unadjusted and adjusted model for the association between CDAI and the risk of HF.

	Model 1		Model 2		Model 3	
	OR (95% CI)	*p* Value	OR (95% CI)	*p* Value	OR (95% CI)	*p* Value
CDAI	0.93 (0.90−0.95)	<.0001	0.95 (0.92−0.98)	.002	0.96 (0.94−0.99)	.02
CDAI						
Q1	1.00 (ref)		1.00 (ref)		1.00 (ref)	
Q2	0.82 (0.66−1.02)	.08	0.87 (0.68−1.12)	.27	0.88 (0.68−1.13)	.30
Q3	0.58 (0.48−0.72)	<.0001	0.69 (0.55−0.87)	.002	0.77 (0.61−0.99)	.04
Q4	0.47 (0.38−0.59)	<.0001	0.61 (0.48−0.79)	<.001	0.68 (0.52−0.89)	.01

*Note*: Model 1: Unadjusted; Model 2: Adjusted with gender + age + education + poverty; Model 3: Adjusted with model 2 + smoke + drink + hypertension + MetS + hyperlipidemia + CKD.

Abbreviations: CDAI, composite dietary antioxidants index; HF, heart failure; MetS, metabolic syndrome; OR, odds ratio.

## 4 DISCUSSION

To our knowledge, this is the first population‐based retrospective cohort study to explore the relationship between CDAI and HF risk. We found that CDAI had a negatively relationship with the risk of HF after adjusting for potential confounders, and people in the higher quartiles of the CDAI score had a reduced the risk of HF compared to those in the lowest quartile of CDAI.

Oxidative stress, one of the most important factors in cell injury, play an important role in the pathogenesis of HF.^5^ Because nutritional supplements play an important role as antioxidants, the focus on the effects of dietary TAC is increasing. Dietary TAC is calculated from the oxygen‐free radiation absorption capacity, and it is associated with a lower risk of HF.^17^ Dietary intakes can regulate the plasma redox and protect from reactive oxygen species. Therefore, high dietary antioxidants may reduce the risk of HF.^18^ The CDAI comprises vitamins (A, C, and E) and nutritional factors (zinc and selenium), as a score of total dietary antioxidants, and is widely used in the clinical situations. High CDAI is a protective factor against depression,^11^ and higher CDAI has been linked to decreased cardiovascular mortality risk.^10^ Vitamin C supplementation increased myocardial contractility and endothelial function.^19^ Vitamin E can also increase endogenous antioxidant levels. In patients with HF, vitamin E supplementation can reduce the risk of developing HF.^20^

## 5 CONCLUSION

In our study, CDAI, a score reflecting the dietary profile of antioxidants, was negatively associated with the risk of HF. Dietary intervention may be an easily modified method to reduce the risk of HF, and further studies involving the underlying mechanisms are needed.

### 5.1 Study limitation

This study had several limitations. First, this was an observational study. Second, the diagnosis of HF was made through a questionnaire survey, making it difficult to assess the severity of HF. In addition, the data on ejection fraction, a subtype of HF, were missing.

## CONFLICT OF INTEREST STATEMENT

The authors declare no conflict of interest.

## Data Availability

The data sets used and/or analyzed during the current study are available from the corresponding author on reasonable request.

## References

[1] Salim VS , Alvaro A , BE J , et al. Heart disease and stroke statistics—2020 update. Circulation. 2020;141:e139‐e596. 31992061 10.1161/CIR.0000000000000757

[2] Groenewegen A , Rutten FH , Mosterd A , Hoes AW . Epidemiology of heart failure. Eur J Heart Fail. 2020;22(8):1342‐1356. 32483830 10.1002/ejhf.1858 PMC7540043

[3] Billingsley HE , Hummel SL , Carbone S . The role of diet and nutrition in heart failure: a state‐of‐the‐art narrative review. Prog Cardiovasc Dis. 2020;63(5):538‐551. 32798501 10.1016/j.pcad.2020.08.004 PMC7686142

[4] Sanches Machado d'Almeida K , Ronchi Spillere S , Zuchinali P , Corrêa Souza G . Mediterranean diet and other dietary patterns in primary prevention of heart failure and changes in cardiac function markers: a systematic review. Nutrients. 2018;10:58. 29320401 10.3390/nu10010058 PMC5793286

[5] Bergamini C , Cicoira M , Rossi A , Vassanelli C . Oxidative stress and hyperuricaemia: pathophysiology, clinical relevance, and therapeutic implications in chronic heart failure. Eur J Heart Fail. 2009;11(5):444‐452. 19346534 10.1093/eurjhf/hfp042

[6] Bertero E , Maack C . Calcium signaling and reactive oxygen species in mitochondria. Circ Res. 2018;122(10):1460‐1478. 29748369 10.1161/CIRCRESAHA.118.310082

[7] Yu YC , Paragomi P , Wang R , et al. Composite dietary antioxidant index and the risk of colorectal cancer: findings from the Singapore Chinese health study. Int J Cancer. 2022;150:1599‐1608. 35001362 10.1002/ijc.33925 PMC8930521

[8] Wright ME . Development of a comprehensive dietary antioxidant index and application to lung cancer risk in a cohort of male smokers. Am J Epidemiol. 2004;160:68‐76. 15229119 10.1093/aje/kwh173

[9] Luu HN , Wen W , Li H , et al. Are dietary antioxidant intake indices correlated to oxidative stress and inflammatory marker levels? Antioxid Redox Signaling. 2015;22:951‐959. 10.1089/ars.2014.6212 PMC4376488 25602689

[10] Wang L , Yi Z . Association of the composite dietary antioxidant index with all‐cause and cardiovascular mortality: a prospective cohort study. Front Cardiovasc Med. 2022;9:993930. 36267633 10.3389/fcvm.2022.993930 PMC9577254

[11] Zhao L , Sun Y , Cao R , Wu X , Huang T , Peng W . Non‐linear association between composite dietary antioxidant index and depression. Front Public Health. 2022;10:988727. 36311643 10.3389/fpubh.2022.988727 PMC9609418

[12] Maugeri A , Hruskova J , Jakubik J , et al. Dietary antioxidant intake decreases carotid intima media thickness in women but not in men: a cross‐sectional assessment in the Kardiovize study. Free Radic Biol Med. 2019;131:274‐281. 30576781 10.1016/j.freeradbiomed.2018.12.018

[13] Dong W , Yang Z . Association of dietary fiber intake with myocardial infarction and stroke events in US adults: a cross‐sectional study of NHANES 2011–2018 [J]. Front Nutr. 2022;9:936926. 35799583 10.3389/fnut.2022.936926 PMC9253671

[14] Grundy SM , Cleeman JI , Daniels SR , et al. Diagnosis and management of the metabolic syndrome: an American Heart Association/National Heart, Lung, and Blood Institute Scientific Statement. Circulation. 2005;112(17):2735‐2752. 16157765 10.1161/CIRCULATIONAHA.105.169404

[15] Kammerlander AA , Mayrhofer T , Ferencik M , et al. Association of metabolic phenotypes with coronary artery disease and cardiovascular events in patients with stable chest pain. Diabetes Care. 2021;44(4):1038‐1045. 33558267 10.2337/dc20-1760 PMC7985425

[16] Kidney Disease: Improving Global Outcomes (KDIGO) Glomerular Diseases Work Group . KDIGO 2021 clinical practice guideline for the management of glomerular diseases. Kidney Int. 2021;100(4S):S1‐S276. 34556256 10.1016/j.kint.2021.05.021

[17] Rautiainen S , Levitan EB , Mittleman MA , Wolk A . Total antioxidant capacity of diet and risk of heart failure: a population‐based prospective cohort of women. Am J Med. 2013;126(6):494‐500. 23561629 10.1016/j.amjmed.2013.01.006

[18] Galarregui C , Zulet M , Cantero I , et al. Interplay of glycemic index, glycemic load, and dietary antioxidant capacity with insulin resistance in subjects with a cardiometabolic risk profile. Int J Mol Sci. 2018;19:3662. 30463312 10.3390/ijms19113662 PMC6275010

[19] Shinke T , Shite J , Takaoka H , et al. Vitamin C restores the contractile response to dobutamine and improves myocardial efficiency in patients with heart failure after anterior myocardial infarction. Am Heart J. 2007;154(4):645.e1‐645.e8. 10.1016/j.ahj.2007.07.005 17892985

[20] Ghatak A , Brar MJS , Agarwal A , et al. Oxy free radical system in heart failure and therapeutic role of oral vitamin E. Int J Cardiol. 1996;57(2):119‐127. 9013263 10.1016/s0167-5273(96)02787-8

